# Anterior cruciate ligament- specialized post-operative return-to-sports (ACL-SPORTS) training: a randomized control trial

**DOI:** 10.1186/1471-2474-14-108

**Published:** 2013-03-23

**Authors:** Kathleen White, Stephanie L Di Stasi, Angela H Smith, Lynn Snyder-Mackler

**Affiliations:** 1 University of Delaware, Biomechanics and Movement Science Program, Newark, DE, USA; 2 The Ohio State University, Sports Health and Performance Institute, Columbus, OH, USA; 3 University of Delaware, Physical Therapy Department, Newark, DE, USA

**Keywords:** Anterior cruciate ligament reconstruction, Neuromuscular training, Return to Sport

## Abstract

**Background:**

Anterior cruciate ligament reconstruction (ACLR) is standard practice for athletes that wish to return to high-level activities; however functional outcomes after ACLR are poor. Quadriceps strength weakness, abnormal movement patterns and below normal knee function is reported in the months and years after ACLR. Second ACL injuries are common with even worse outcomes than primary ACLR. Modifiable limb-to-limb asymmetries have been identified in individuals who re-injure after primary ACLR, suggesting a neuromuscular training program is needed to improve post-operative outcomes. Pre-operative perturbation training, a neuromuscular training program, has been successful at improving limb symmetry prior to surgery, though benefits are not lasting after surgery. Implementing perturbation training after surgery may be successful in addressing post-operative deficits that contribute to poor functional outcomes and second ACL injury risk.

**Methods/Design:**

80 athletes that have undergone a unilateral ACLR and wish to return to level 1 or 2 activities will be recruited for this study and randomized to one of two treatment groups. A standard care group will receive prevention exercises, quadriceps strengthening and agility exercises, while the perturbation group will receive the same exercise program with the addition of perturbation training. The primary outcomes measures will include gait biomechanics, clinical and functional measures, and knee joint loading. Return to sport rates, return to pre-injury level of activity rates, and second injury rates will be secondary measures.

**Discussion:**

The results of this ACL-Specialized Post-Operative Return To Sports (ACL-SPORTS) Training program will help clinicians to better determine an effective post-operative treatment program that will improve modifiable impairments that influence outcomes after ACLR.

**Trial registration:**

Randomized Control Trial NIH 5R01AR048212-07. ClinicalTrials.gov: NCT01773317

## Background

Anterior cruciate ligament reconstruction (ACLR) is standard practice for individuals that desire to return to high-level activities, but excellent outcomes are not as commonplace as previously reported [1-5]. Currently, success after ACLR is measured using return-to-sport rates, but second ACL injuries are not only common, but devastating, and have worse outcomes than primary ACLR [6-8]. Quadriceps weakness [9-11], abnormal movement patterns [4,12-16] and below normal knee function [17] are characteristic of athletes in the months following ACLR and often persist up to two years in spite of extensive rehabilitation. Neuromuscular training focusing on restoring limb symmetry and improving knee function using sports-related movements may reduce aberrant movement patterns which are predictive of second injury risk [4,12,18,19].

Risk of a second ACL injury is highest during the first year that athletes return to sports after primary ACL reconstruction [4,13,20-22]. Young females are 16 times more likely to sustain a second ACL injury after primary ACLR and the amount of participation time in high-level activities further increases this risk [23]. Risk to the contralateral limb is higher (5-24%) than the operated limb (4-15%) [4,23-28], suggesting deficits of the involved limb are not exclusively related to re-injury. Altered neuromuscular and biomechanical movement patterns are present bilaterally in response to injury and reconstruction which fails to resolve with post-operative rehabilitation. A neuromuscular training program focused on maximizing performance after ACLR may reduce the risk of a second ACL injury.

Despite current evidence-based post-operative guidelines [29-32], quadriceps strength deficits [9-11], altered biomechanics [4,13-16,33] and poor knee function [17] are reported six months and one year after surgery. International Knee Documentation Committee 2000 subjective knee form (IKDC 2000) scores continue to improve up to one year after surgery suggesting optimal knee function has not been met [17]. Despite clearance for return to sport activities by surgeons and rehabilitation specialists, quadriceps strength deficits of the involved limb compared to the uninvolved limb still exist, and movement asymmetries continue to persist [10,11,14,16].

One year after surgery only 67% of patients have attempted some sort of training or sport activity; males are more likely than females to attempt full return to sport [34]. Individuals often do not return to their pre-injury activity level for a variety of reasons; fear of re-injury being a large contributing factor [35-37]. Patients in the medium to long term after surgery (two to seven years) that have returned to their pre-injury activity level were less likely to be fearful of re-injury during athletic participation than those that had not returned to their pre-injury level [37]. Females were more fearful with poor environmental conditions during athletic participation than their male counterparts [37].

Neuromuscular training, consisting of destabilizing perturbations to both the involved and uninvolved lower extremities, has been an effective means of enhancing functional outcomes after ACL injury compared to strength training [12,38]. Neuromuscular training programs such as perturbation training (PERT) [39] before surgery reduce gait asymmetries in female non-copers [18]. After surgery, non-copers who received pre-operative PERT demonstrated improved knee excursion symmetry during gait compared to patients who received strength training [12]. However, regardless of pre-operative intervention, aberrant movement patterns persisted up to two years after surgery [14]. Pilot data from our lab strongly suggests that utilizing this neuromuscular training program after surgery will be an effective means of improving both short term outcomes (6 months), when clearance to return to sport often occurs, and medium term outcomes (1-2 years) after surgery. Successful primary ACL prevention programs utilize a combination of balance, plyometric and strengthening exercises to decrease ACL injury risk. Similarly, our ACL-Specialized Post-Operative Return To Sports (ACL-SPORTS) Training will incorporate dynamic prevention exercises and quadriceps strengthening exercises that promote symmetrical joint loading and abate abnormal movement patterns. A post-operative intervention incorporating these elements with the addition of PERT may be effective in resolving residual impairments after surgery.

The purpose of this study is to determine the effects of this ACL-SPORTS Training program on joint loading, biomechanics, and clinical and functional measures of level 1 and 2 athletes after ACLR. This body of work will further explain in detail each component of the training program as well as the methodology of this single blinded randomized control trial.

### Hypotheses

Subjects who receive standard care plus PERT after surgery will demonstrate: 1) symmetrical knee joint loading, 2) symmetrical movement patterns, 3) improved clinical and functional outcomes and 4) improved knee function compared to subjects who receive standard care. Additionally, subjects who receive standard care plus PERT will have a higher return to pre-injury level rates in the short to medium term (6 months -2 years) compared to subjects who receive standard care.

## Methods/Design

This study is a single-assessor blinded, parallel design randomized control trial that follows the CONSORT guidelines for non-pharmacological treatment studies [40]. Additional information about this study can be found at: Clinicaltrials.gov (Identifier: NCT 01773317).

### Participants

Eighty level 1 and 2 athletes (40 men, 40 women) between the ages of 13 and 55 that have undergone an isolated, unilateral ACL reconstruction will be recruited for this study. Recruiting will be done primarily through the University of Delaware Physical Therapy Clinic. Additional recruitment will consist of newspaper advertisements as well as speaking with local surgeons and rehabilitations specialists. Athletes will be eligible for study enrollment if they were participants in level 1 or 2 activities [1] ≥ 50 hrs/year at the time of their injury, plan to return to their pre-injury level of activity, are ≥ 12 weeks after surgery, demonstrate ≥ 80% quadriceps strength index and minimal knee joint effusion [41].

### Exclusion criteria

**Figure 1:**
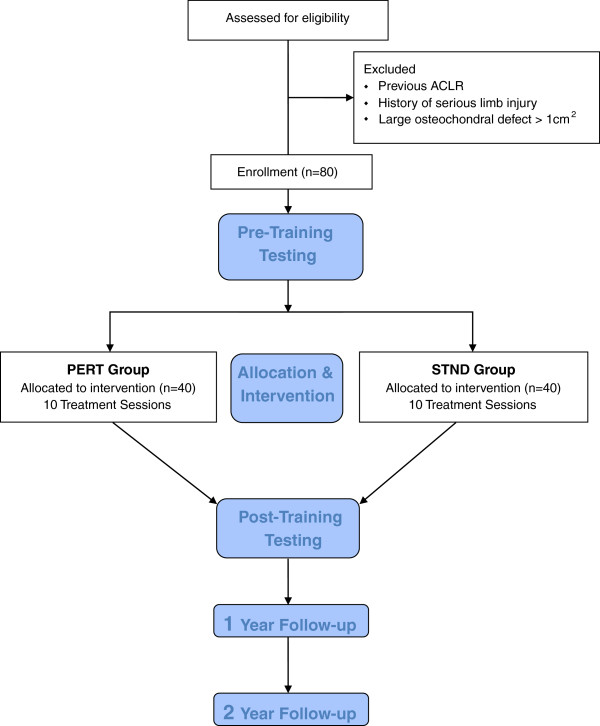
CONSORT flow diagram of study proctol.

Subjects will be excluded if: (i) not regular participants in level 1 or 2 activities (< 50 hrs/yr), (ii) > 10 months after ACLR, (iii) history of previous ACLR, (iv) history of serious ipsilateral or contralateral limb injury (i.e. Tibial fx), or (v) large osteochondral defect > 1 cm^2^ (Figure 1. CONSORT Flow Diagram of Study Protocol).

### Procedure

Once a patient has consented to be contacted by research staff, eligibility will be determined from patient chart review and patient dialogue. All testing measures will be performed at the University of Delaware Physical Therapy Clinic by the same assessor, blinded to group assignment. Enrolled study participants will complete initial baseline testing and 10 treatment sessions followed by post-training, 1 year and 2 year follow-up testing sessions. Testing will be completed no more than two weeks prior to initiation and two weeks after the completion of the 10 training sessions. Ethical approval has been obtained from the University of Delaware Human Subjects Review board. All participants will provide written informed consent to all research testing procedures.

### Randomization and allocation concealment

Enrolled subjects will be randomized to a perturbation treatment group (PERT) or a standard treatment group (STND). A statistical random number generator will be used to generate a randomization list, stratified by gender in which an equal number of female and male subjects will be assigned to each treatment group. The research coordinator will generically label the treatment assignments to group A and B to ensure blinding is maintained. This is a single-blinded study in that individuals collecting, recording and analyzing these data will be blinded to group assignment along with the primary investigator and biostatistician. Both the treating physical therapist and the patient will not be blinded to group assignment.

### Interventions

Treatment will be completed at the University of Delaware Physical Therapy clinic by the licensed staff therapists. The therapists have an average of 6.5 yrs experience (range 1 – 20 yrs). All therapists will participate in a training session involving discussion of the treatment program and how to manage changes in effusion, complaints of muscle soreness and still effectively complete the training program. A detailed protocol with visual aids and descriptions will be provided. Once training has been initiated procedural reliability will be completed by an unblinded investigator to ensure that the intervention is properly executed. An unmasked physical therapy clinic liaison will utilize a procedural reliability check list to review three treatment sessions for the first five subjects in each arm of the study. After that each subject’s chart will be reviewed once (Additional file 1). Procedural reliability less than 85% will result in contacting the treating therapist to remedy the situation. Any additional questions regarding the training program will be intercepted by the clinic liaison to ensure blinding of those responsible for data collection is maintained.

The training protocol consists of 10 training sessions with treatment delivered by a licensed physical therapist, regardless of group allocation. Biomechanical and functional data will be collected prior to initiation of the 10 training sessions, after completion of the sessions, and 1 and 2 years after surgery. The training program consists of a series of “prevention exercises”, quadriceps strengthening exercises, agility drills and either PERT training for individuals in the perturbation group or a control exercise for individuals in the STND care group. Details of these exercises are explained in further detail below.

Prevention exercises: A combination of balance, plyometric and strengthening exercises are effective in preventing initial ACL injury [30,42]. Plyometric training improves landing biomechanics in females [43] and decreases ACL injury rates [44]. Balance training not only improves lower extremity strength, but eliminates limb asymmetries [45], which are potential risk factors for ACL injury [4,46]. Strengthening programs alone do not reduce the number of ACL injuries [47], however when combined with plyometric training there is a significant reduction in ACL injuries, specifically female athletes [44]. Established injury prevention protocols were modified to develop the “prevention exercises” for this study (Table 1). Plyometric and balance exercises include triple single-legged hops, tuck jumps and box drops; strengthening exercises include nordic hamstring curls and squats with hip abduction resistance. Triple single-legged hops are done consecutively, forward and backwards as well as laterally. The initial drill will be completed by hopping over a line on the floor and progressed to hopping over 2 inch cups and ultimately 6 inch hurdles. Progression and cueing will be given by the treating therapist as per the patient’s ability level with a training protocol as a guide (Table 1). Tuck jumps will not be completed until treatment sessions 7-10 to ensure that patients are able to tolerate jump landings safely and successfully. Box drops begin bilaterally and progress to unilateral jumps (involved limb to involved limb). Focus on mechanics during double limb tasks initially allows for an effective progression to single limb tasks [48]. The box height will be progressively increased by the treating therapist as per the patient’s ability level using the training protocol as a guide (Table 1). This task will be completed in front of a mirror for visual feedback while the therapist provides verbal cues. Proper mechanics will be required including symmetrical limb takeoff and landing for bilateral tasks, good trunk control and neutral frontal plane knee alignment during takeoff and landing for bilateral and unilateral tasks. Nordic hamstring exercises will be completed on a low mat table with the therapist stabilizing the patient’s ankles. Initially this eccentric hamstring activity will be done to about 30-45 degrees of knee flexion and repetitions as well as knee flexion angle will be progressed over the 10 training sessions. Resisted squat exercise will be done with a thera-band around the patient’s knees to facilitate hip abduction. The resistance of the thera-band will increase as tolerated by the patient and additional upper extremity tasks such as a ball toss will be added to increase the difficulty of the task and challenge the patient. These exercises will be executed with the patient wearing a rigid functional knee brace if one has been prescribed by the surgeon. If a patient will be returning to sport without a knee brace then all testing and training sessions will be done without a brace.

**Table 1:** ACL-SPORTS training protocol (2 times/wk)

	**Session 1-3**		**Session 4-6**	**Session 7-10**
**Nordic Hamstrings**	Partial 2 x 5		Partial 3 x 5	Partial 3 x 5
Kneeling on mat table, therapist stabilizing feet	(~30-45°)		(~30-45°)	(>60°)
**Standing Squat**	Session 1: 3x10 with focus on proper technique	3 x 10	3 x 10	
Must squat to knees at 90 degrees, tapping chair/table/box with gluts		Add t-band around knees	progress t-bands to black	X
Drop jumps**	3 x 10 BLE’s to BLE’s		3 x 10 BLE’s to involved limb	3 x 10 Involved limb to involved limb off box
In front of mirror, monitor proper form with landing	Jump off appropriate height (4-6-8 inch)			
			Jump off appropriate height (4-6-8 inch)	Jump off appropriate height (4-6-8 inch)
Triple single leg hopping**	Forward/backward x10*		Forward/backward x15*	Forward/backward x15*
	Side to side x10*		Side to side x15*	Side to side x15*
	No object		Add low object to jump over (2 inch cups)	Increase height of object, appropriate for the pt. (4 inch cups or 6 inch hurdles)
This is for proper landing, NOT distance				
Tuck jumps**				2 sets, 10-20 sec
Proper form knees to 90°	X		X	Progress to 3 sets, 20-30 seconds each

**Brace worn if surgeon requires post-op functional brace for RTS activities. *1 rep= 3 consecutive hops forward, 3 hops backward or 3 consecutive hops laterally.

Quadriceps strengthening: The results of baseline testing measures will be used to determine the patient’s need for quadriceps strengthening during the 10 training sessions. A patient that demonstrates > 90% quadriceps strength index (involved limb strength/uninvolved limb strength × 100) will not be required to complete quadriceps strengthening exercises during training, but they may continue their prior gym program. All other patients with 80-90% quadriceps strength index will complete three quadriceps strengthening exercises during three of the first six training sessions including but not limited to, lateral step downs, leg press, LAQ and isokinetic strengthening. After the 6^th^ training session the patient will be given a home strengthening program because of the progressive nature of the program and the advanced level of tasks during the last 4 sessions.

Agilities: Agility drills will be completed as per the University of Delaware guidelines. Drills will be initiated at 50% maximum effort and progressed to 100% effort and maximum speed over the 10 training sessions. Three to four agilities drills will be completed at each training session including forward/backward running, side shuffles, cariocas, figure eight’s, circles and 90 degree turns. The treating therapist will determine which agility drills to use based on the patients sports participation and ability level. Progression of these drills will include eliminating linear drills, adding more advanced multidirectional drills and utilizing a ball consistent with the patient’s sport of participation.

Perturbation training group: Patients randomized to the PERT group will complete additional PERT training as per Fitzgerald et al. [38]. PERT training is a neuromuscular training program that includes a series of progressive perturbations on unstable surfaces in both bilateral and unilateral stance. These are progressed as per patient tolerance in both magnitude and speed. Verbal distraction as well as the addition of simultaneous upper extremity or lower extremity tasks with perturbations will be used to target the individuals sport and challenge the athlete.

**Figure 2:**
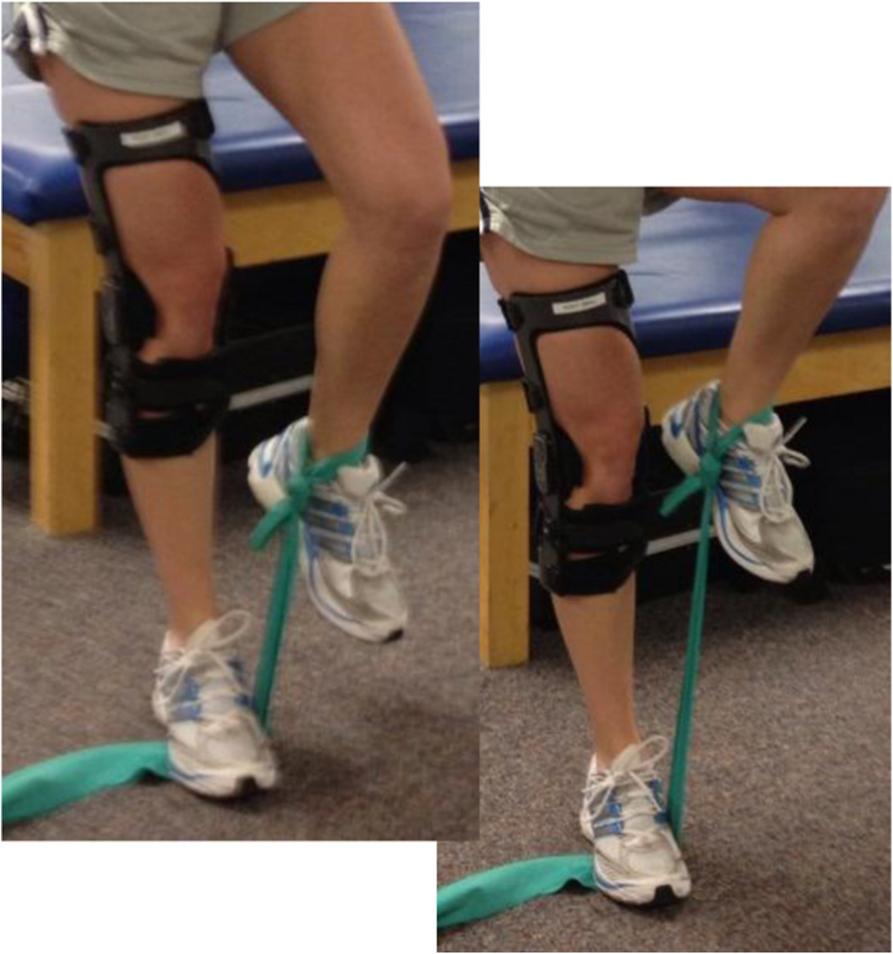
STND Treatment Group Additional Exercise.

Standard treatment group: Patients in the STND group will complete an additional single leg balance task with added hip flexor resistance (Figure 2). This exercise will not be progressed to unstable surfaces to ensure that similar neuromuscular effects are not seen in this group. This exercise will only increase in duration and thera-band resistance (Table 2). All treatment sessions, regardless of group, will take about 1.5-2 hours to complete.

**Table 2:** STND treatment group additional exercise

**Control subjects**	**Session 1-3**	**Session 4-6**	**Session 7-10**
Single Leg Balance	3 x 30 sec	3 x 45 sec	3 x 1 minute
(**Do Not** “progress” with unstable surfaces or ball toss or perturbation)	(Level Ground)	Add sham progression: (stabilize t-band with standing leg, complete hip flexion with tband around ankle)	same

**Figure 3:**
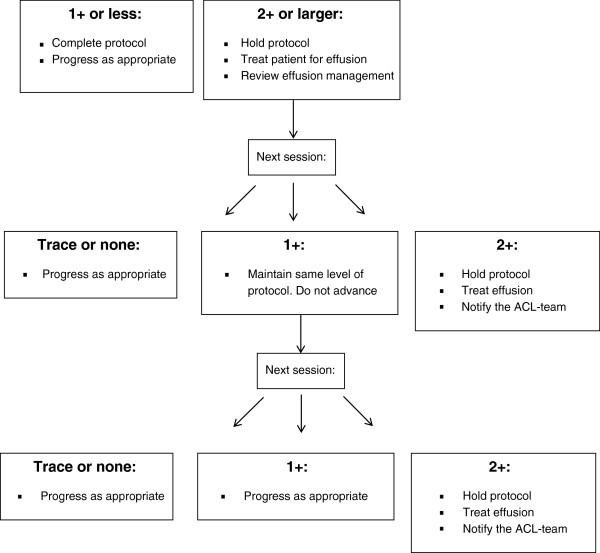
ACL-SPORTS training effusion protocol.

**Figure 4:**
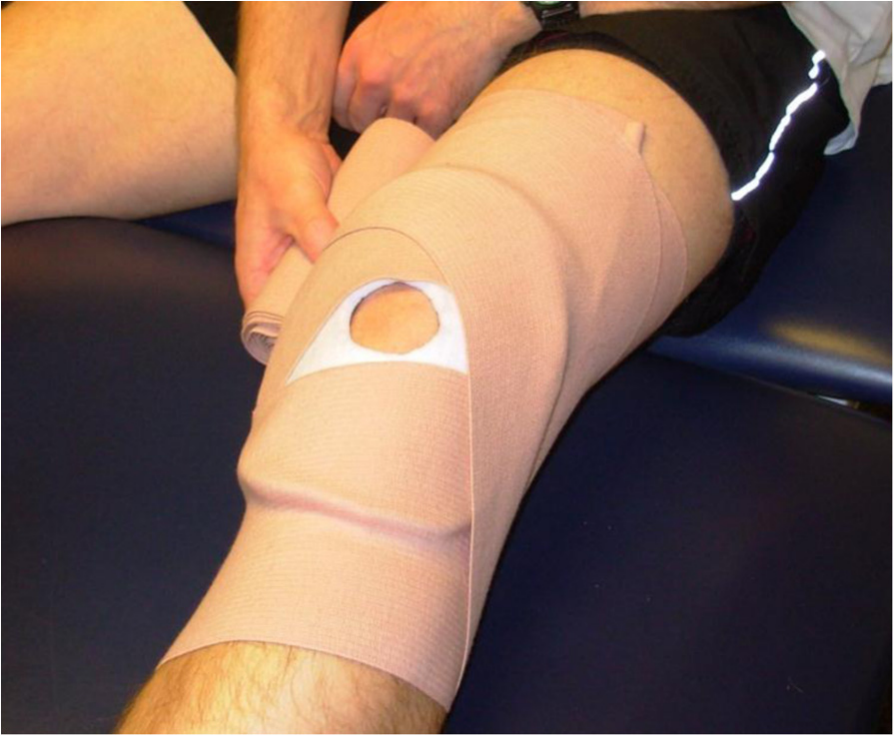
Donut with compression wrap for effusion management.

The expertise of the treating therapists will determine if any task is unsafe for the patient and should be held from the protocol at any point. If a patient develops increased knee joint effusion [41] or additional complaints of pain, a clinical decision making protocol has been established as a guide for the therapists to determine how to modify the training program (Figure 3). If a patient present with a 2+ effusion at any point during the training program the training protocol will be held and the patient will be treated with effusion management including retrograde massage, ice and elevation. The patient will be educated on proper effusion management techniques such as keeping the knee wrapped with a donut and compression wrap (Figure 4), as well as icing several times a day with the leg elevated. At the following session if the effusion has decreased to a 1+ the training will resume at the same level of difficulty, if the patient has trace or no effusion then the training program will be progressed accordingly. Conversely, if the patient continues to demonstrate a 2+ effusion the training will be held, the patient will be treated accordingly for effusion and the research team will be notified. Additional complications that occur throughout training will be treated as needed by the treating therapist. If additional symptoms or impairments are limiting completion of the training program the principle investigator will be notified. If the patient cannot resume the training program for any reason the training will be terminated and post-training data will be collected. The patient will continue to be treated accordingly for their impairments.

### Outcomes measures

The primary outcome variables of interest for this study will include gait biomechanics, clinical outcome measures and knee joint loading.

Gait biomechanics will be assessed using a 3D motion capture system (VICON, Oxford Metrics Ltd., London, England) sampled at 120 Hz. Twenty static retro-reflective markers will be placed on the pelvis and lower extremities to identify limb segments. An embedded force plate (Bertec, Worthington, OH) will simultaneously collect kinetic data and used to determine timing variables during the gait cycle. Five walking trials will be collected for each limb while patients maintain a self-selected walking speed with ± 5% variability. These data will be post-processed using rigid body analysis and inverse dynamics with custom software programming (Visual3D, C-Motion, Inc., Germantown, MD, USA; LabVIEW 8.2, National Instruments Corp., Austin, TX, USA). Variables will be lowpass filtered at 6 Hz and 40 Hz. Initial contact and toe off will be determined using a 50 N force plate threshold. All walking trials will be normalized to 100% of stance before being averaged for statistical analysis. Hip and knee joint angles, moments and excursions will be evaluated between limbs in both the sagittal and frontal plane.

Clinical outcome measures will include quadriceps strength index, single-legged hop test measures and patient reported outcome measure. Quadriceps strength will be measured using a maximal voluntary isometric contraction (MVIC) with a burst superimposition technique [49]. Activation deficits and isometric quadriceps strength will be measured using an electromechanical dynamometer (KIN-COM, Chattanooga Corp., Chattanooga, TN). Patients will be seated in an upright position with the hip and knee flexed to 90 degrees. Testing will be completed on the uninvolved limb followed by the involved limb. A quadriceps index (QI) will be calculated as the quotient of the involved quadriceps MVIC to the uninvolved quadriceps MVIC multiplied by 100. The single-legged hop test measures [50] will consist of four hop tests in which each test will be administered as the uninvolved limb followed by the involved limb for the single hop for distance, crossover hop for distance, the triple hop for distance and 6-meter timed hop tests. A limb symmetry index (LSI) will be calculated from the average of two trials as the involved limb hop distance divided by the uninvolved limb hop distance multiplied by 100. The 6-meter timed hop will be calculated as the uninvolved limb hop time divided by the involved limb hop time multiplied by 100. Patient reported outcome measures will be completed after all objective clinical measures have been collected. The Knee Outcome Survey-Activities of Daily Living Score (KOS-ADLS) and the Global Rating Scale of Perceived Knee Function (GRS) will be used to determine the patients perceived knee function. A strict return to sport criteria, established by Fitzgerald et al. [38], requires the patient to achieve ≥ 90% on the following measures: QI, all 4 single-legged hop tests, KOS-ADL’s and GRS. Patients will be required to meet these criteria after training to allow for progressive return to sport activities. If patients do not meet these criteria after training they will be repeatedly tested every 2-4 weeks until all measures are met prior to returning to sport activity. The ACL-Return to Sport after Injury (ACL-RSI) has been validated to measure fear in patients after ACLR. This patient reported outcome measure will be used to objectively measure patient fear in the short to medium term (6 months -2 years).

#### Joint loading

Electromyography (EMG)-driven musculoskeletal modeling will be used to estimate muscle forces from EMG muscle data during walking trials [51]. Anatomical modeling of the pelvis and lower limbs will be scaled initially for each subject. The model will then be calibrated based on muscle parameters used to determine the EMG-to-force relationship. Through iterative adjustments, the muscle parameters will result in strong agreement with the sagittal plane net moments calculated from forward and inverse dynamics. Once the ideal model is determined, the muscle forces will be predicted from mathematical calculations from recorded EMG for three walking trials and converted to muscle force. A frontal plane moment balancing algorithm [52] will be used to calculate medial and lateral compartment contact forces. The knee adduction moment will be calculated using inverse dynamics and will be expressed about each contact point in the medial and lateral compartments. A balance of contact forces and muscle forces at each contact point will be summated to express the contact forces in the each compartment as well as the total joint forces.

### Secondary outcome measures

Return to sport rates, re-injury rates and return to pre-injury level of activity rates will be evaluated 1 and 2 years after surgery. Electromyography measures will be collected simultaneously with gait variables and will be used to further analyze muscle timing, co-contraction and activation patterns before and after the intervention as well as 1 and 2 years after surgery. IKDC 2000, Tampa Scale of Kinesiophobia (TSK-11), Knee Injury and Osteoarthritis Outcome Score (KOOS) including all 5 subsets and the Marx Activity Rating Scale (MARS) are additional patient reported outcome measures that will be collected at all-time points.

### Sample size

Minimal clinically importance differences (MCID) for sagittal plane gait variables have previously been established [18]. A power analysis with β = 0.20, α = 0.05 and a medium effect size (0.3) determined that 72 subjects would be needed to detect differences between groups based on MCID’s. To account for a 10% patient drop out a total of 80 subjects will be enrolled in this study. Forty patients will be in each group dichotomized by gender.

### Data and statistical analysis

Differences between groups will be analyzed using an analysis of covariance (ANCOVA) for biomechanical gait measures and an analysis of variance (ANOVA) will be used for clinical variables. Group assignment will be blinded to the researcher using A and B variables. Assumptions of ANOVA testing will be confirmed prior to statistical analysis. Training group randomization will be used as the between-subjects factor with a within-subjects factor of time. A significance level of p < 0.05 will be set a priori.

### Timeline

Human subjects review board approval was obtained in July 2011 from the University of Delaware Institutional Review Board and recruitment and training was initiated in November 2011. A projected 25 patients will be enrolled in the study within the first year followed by 30 and 25 patients respectively in the subsequent years. Final enrollment is planned to be completed by November 2014 and final data collection and analysis is planned to be completed by November 2016.

## Discussion

Both short and long term outcomes after ACLR are poorer than previously reported in high-level athletes [1-5]. The explanation of these low return to sports rates appears to be multi-factorial, but may be heavily influenced by lower perceived level of knee function and fear of re-injury [37,53,54]. The relationship of physical performance measures to these subjective evaluations and perceptions of ability are unknown. The aim of this project is to compare the outcomes of two different return to sport training programs in order to establish best-practice guidelines for this high-risk population.

Initial ACL injury rates continue to be elevated and subsequent re-injury rates are even higher despite the positive evolution of post-operative rehabilitation protocols [4,23-28]. Quadriceps weakness, abnormal movement patterns and decreased knee function persist after athletes have returned to sports, supporting the need for a bilateral, neuromuscular training program to promote improved outcomes after ACLR [4,9-17]. Our program was compiled from the latest evidence emphasizing prevention exercises, quadriceps strengthening and perturbation training as a plausible mechanism by which clinicians can maximize post-operative function and reduce second ACL injury risk.

Our study is the first randomized control trial to evaluate the effects of a post-operative intervention program on joint loading, gait biomechanics and clinical outcome measures. Implementing this program in our physical therapy clinic with therapists who have years of expertise executing research protocols allows us to make this post-operative training program generalizable to clinical practice while maintaining the rigor of scientific research. Our subjects will represent several different orthopedic surgeons with a variety of graft types which will allow us to evaluate additional factors outside of our rehabilitation protocol. Our criterion to implement training is based on an array of evidence based clinical measures rather than time based measures (i.e. 6 months) or surgical findings (i.e. bone bruise, meniscus repair) [29,30,38,55]. Group randomization by gender will ensure that effects of treatment are adequately captured. Blinding of researchers collecting these data allows for unbiased reporting of results.

Through this ACL-SPORTS Training program we will be able to better evaluate the effects of neuromuscular training after surgery on knee joint loading, gait biomechanics and clinical outcome measures for these athletes. These variables are modifiable factors reported in the literature and most commonly utilized in clinical practice. Results of this study will allow us to develop future treatment plans to maximize functional outcomes in the short and long term after ACLR.

## Abbreviations

ACL: Anterior cruciate ligament; ACLR: Anterior cruciate ligament reconstruction; IKDC 2000: International knee documentation committee 2000; PERT: Perturbation; ACL-SPORTS: Anterior cruciate ligament- specialized post-operative return-to-sports; STND: Standard; MVIC: Maximal voluntary isometric contraction; QI: Quadriceps index; LSI: Limb symmetry index; KOS-ADLS: Knee outcome survey-activities of daily living score; GRS: Global rating scale; ACL-RSI: Anterior cruciate ligament- return to sports after injury; EMG: Electromyography; TSK-11: Tampa scale of Kinesiophobia; KOOS: Knee injury and Osteoarthritis outcome score; MARS: Marx activity rating scale; MCID: Minimal clinically important differences; ANCOVA: Analysis of covariance; ANOVA: Analysis of variance

## Competing interests

The authors declare that they have no competing interests.

## Authors’ contributions

KW participated in developing the protocol, instructing the physical therapy staff on the treatment protocol, patient recruitment, research testing, contributed to trial registration on ClinicalTrials.gov, and drafted and critically revised this manuscript for important intellectual content. SLD contributed to the original idea of the study, developed a manual of operating procedures and critically revised this manuscript for important intellectual content. AHS participated in patient recruitment, procedural reliability, edited the treatment protocol, edited the procedural reliability forms and critically revised this manuscript for important intellectual content. LSM is the primary investigator of the randomized control trial and is responsible for the original idea of the study, registering the study on ClinicalTrials.gov, and critically revised this manuscript for important intellectual content. All authors read and approved the final manuscript.

## Authors’ information

KW, PT, DPT: KW is a PhD student and research assistant at the University of Delaware in the Biomechanics and Movement Science program. She is also a teaching assistant for several classes in the Department of Physical Therapy and is a per diem physical therapist at the University of Delaware Physical Therapy Clinic.

SLD, PhD, PT: SLD is currently conducting research on the recovery of function in athletes with ACL injuries and femoroacetabular impingement (FAI). She is currently funded by the Sports Physical Therapy Section of the American Physical Therapy Association for her work with FAI. SLD is also a part-time physical therapist at the Ohio State University Sports Medicine Physical Therapy Clinic.

AHS, PT, DPT, OCS, SCS, ATC: AHS is a senior staff physical therapist in the Sports and Orthopaedic Physical Therapy Clinic as the University of Delaware. She is the physical therapy liaison overseeing the execution of the treatment protocol and completing procedural reliability for this study.

LSM, PT, ScD, ATC, FAPTA: LSM, an alumni distinguished professor at the University of Delaware, is faculty in the Physical Therapy Department, Kinesiology and Applied Physiology Department, Biomedical Engineering Department and the Biomechanics and Movement Science program. She is also the Academic Director of the University of Delaware Physical Therapy Clinic and the Director of the Residency Programs of the Physical Therapy Clinic at the University of Delaware.

## Pre-publication history

The pre-publication history for this paper can be accessed here:

http://www.biomedcentral.com/1471-2474/14/108/prepub

## Supplementary Material

Additional file 1 **ACL-SPORTS Training.** Treatment Procedural Checklist. Click here for file
