# Supplementary material for: Anterior cruciate ligament- specialized post-operative return-to-sports (ACL-SPORTS) training: a randomized control trial
Source: BMC Musculoskelet Disord. 2013 Mar 23;14:108. doi: 10.1186/1471-2474-14-108 (PMC3617067; doi:10.1186/1471-2474-14-108)
Supplement: Additional file 1 — ACL-SPORTS Training. Treatment Procedural Checklist. [file 1471-2474-14-108-S1.pdf]

Additional File 1.

ACL-SPORTS Training
Treatment Procedural Checklist

Patient Name:____
Treatment Date of Reliability: ______________
Session # ________

	Y	N	NA
**Quadriceps Strength**			
(If strength < 90%)			
Quadriceps Strengthening Exercise #1 (________________)	____	____	____
* Dosage documented	____	____	____
Quadriceps Strengthening Exercise #2 (__________________)	____	____	____
* Dosage documented	____	____	____
Quadriceps Strengthening Exercise #3 (__________________)	____	____	____
* Dosage documented	____	____	____
**ACL-Sports Exercises**			
Nordic Hams	____	____	____
Standing squat	____	____	____
Drop Jumps	____	____	____
SL hopping	____	____	____
Tuck Jumps	____	____	____
**Agilities**			
Fwd Jog to Backpedal	____	____	____
Side Shuffle	____	____	____
Cariocas	____	____	____
45° cuts	____	____	____
90° cuts	____	____	____
Direction changes on command	____	____	____
Other	____	____	____

Control group only

SLB	per protocol	____	____	____

Perturbation Training group only

Rollerboard	____	____	____
Rollerboard/Platform			
(Involved)	____	____	____
(Uninvolved)	____	____	____
Tiltboard (a/p)	____	____	____
Tiltboard (m/l)	____	____	____
Tiltboard (diag)	____	____	____

Number of Y responses_______

Number of Applicable Treatments_______

Score = # of Y / # applicable treatments x 100 = _______

(Needs to be ≥ 85%)
